# The emerging role of neutrophil extracellular traps in fungal infection

**DOI:** 10.3389/fcimb.2022.900895

**Published:** 2022-08-12

**Authors:** Chuting Liang, Ni Lian, Min Li

**Affiliations:** ^1^ Jiangsu Key Laboratory of Molecular Biology for Skin Diseases and Sexually Transmitted Infections (STIs), Chinese Academy of Medical Sciences and Peking Union Medical College, Institute of Dermatology, Nanjing, China; ^2^ Center for Global Health, School of Public Health, Nanjing Medical University, Nanjing, China

**Keywords:** neutrophil, neutrophil extracellular trap, fungi, mycosis, Candida, Aspergillums

## Abstract

Fungal infections are global public health problems and can lead to substantial human morbidity and mortality. Current antifungal therapy is not satisfactory, especially for invasive, life-threatening fungal infections. Modulating the antifungal capacity of the host immune system is a feasible way to combat fungal infections. Neutrophils are key components of the innate immune system that resist fungal pathogens by releasing reticular extracellular structures called neutrophil extracellular traps (NETs). When compared with phagocytosis and oxidative burst, NETs show better capability in terms of trapping large pathogens, such as fungi. This review will summarize interactions between fungal pathogens and NETs. Molecular mechanisms of fungi-induced NETs formation and defensive strategies used by fungi are also discussed.

## Introduction

Pathogenic fungi have a significant effect on human health by leading to either superficial or invasive infection or both (Köhler et al., 2014). Skin and mucosal infections are the sites of most fungal diseases. Invasive infections are of lower morbidity, yet they kill 1.5 million individuals annually despite several available antifungal drugs (Schwartz, 2004; Brown et al., 2012; Vallabhaneni et al., 2016; Fisher et al., 2018). *Candida albicans, Aspergillus fumigatus*, and *Cryptococcus neoformans* are the top species leading to fungal-associated deaths (Kim, 2016). Significantly, the efficiency of current antifungals is compromised by the emergence of drug tolerance in pathogen populations and additional drug-resistant species, such as *C. auris* and *C. glabrata* (Rhodes and Fisher, 2019; Latgé and Chamilos, 2019; Coste et al., 2020; Lee et al., 2021). Drug–drug interactions, toxicity, and poor bioavailability are also responsible for the limitations of traditional antifungal agents (Stewart and Paterson, 2021). It is widely accepted that strengthening the fungicidal capability of the host immune system may provide a prospective therapeutic strategy (Lee et al., 2021). Therefore, insight into the mutual effects between fungal pathogens and the immune system may provide new ideas for clinical therapy of fungal infections.

Polymorphonuclear leukocytes (neutrophils), one kind of phagocyte, play a crucial role in the innate immune system. As the primary effecter in host defense, their role in combating fungal pathogens is generally recognized. Neutrophils defend against fungal infection by secreting antimicrobial peptides, cytokines, and chemokines, depriving fungal spores of essential nutrients, engulfing fungal spores, and releasing neutrophil extracellular traps (NETs) (Brinkmann et al., 2004; Pathakumari et al., 2020). NETs are netlike structures and capture and kill fungal hyphae that are too large to undergo phagocytosis. Since being discovered in 2004, NETs have received increasing scrutiny although studies mainly focus on the role of NETs in bacterial infections. Their role in fungal infections is usually overlooked. In this review, the molecular mechanisms of NET formation is described. The emerging role of NETs in fungal infections and fungal responses are summarized. Additionally, controversy in this particular field will be discussed.

## Neutrophil extracellular traps and NETosis

NETs are extracellular reticular structures released from activated neutrophils and consist of both nuclear and granular components. The nuclear components are composed of histones and highly depolymerized chromatin. Most chromatin in NETs is derived from nuclear DNA, but current studies reveal that mitochondrial DNA is also involved (Lood et al., 2016). The granular components include granule proteins, such as like myeloperoxidase (MPO), neutrophil elastase (NE) and calprotectin in addition to cytosolic proteins, such as actin and α actinin, which provide microbicidal activity (Urban et al., 2009). NETs are related to many diseases and can lead to adverse cardiovascular events (Bonaventura et al., 2020), tumor development and metastasis (Masucci et al., 2020), and autoimmune diseases (Lee et al., 2017). In terms of infectious diseases, the formation of NETs was initially described in bacterial infections. Subsequently, NET formation stimulated by fungi, viruses, and parasites has also been demonstrated. Intriguingly, NETs retain antimicrobial activity when they encounter large pathogens, such as like *C. albicans* hyphae and *Leishmanis infantum* promastigotes (Gabriel et al., 2010; Brinkmann et al., 2004; Saitoh et al., 2012; Wardini et al., 2019; Arcanjo et al., 2020). Nevertheless, NETs act as a double-edged sword in the infectious milieu. When NETs capture and neutralize pathogens, they also lead to collateral damage by intensifying inflammatory cellular responses, such as in pulmonary infections induced by COVID-19 and *A. fumigates* (Ellett et al., 2017; Arcanjo et al., 2020).

**Figure 1:**
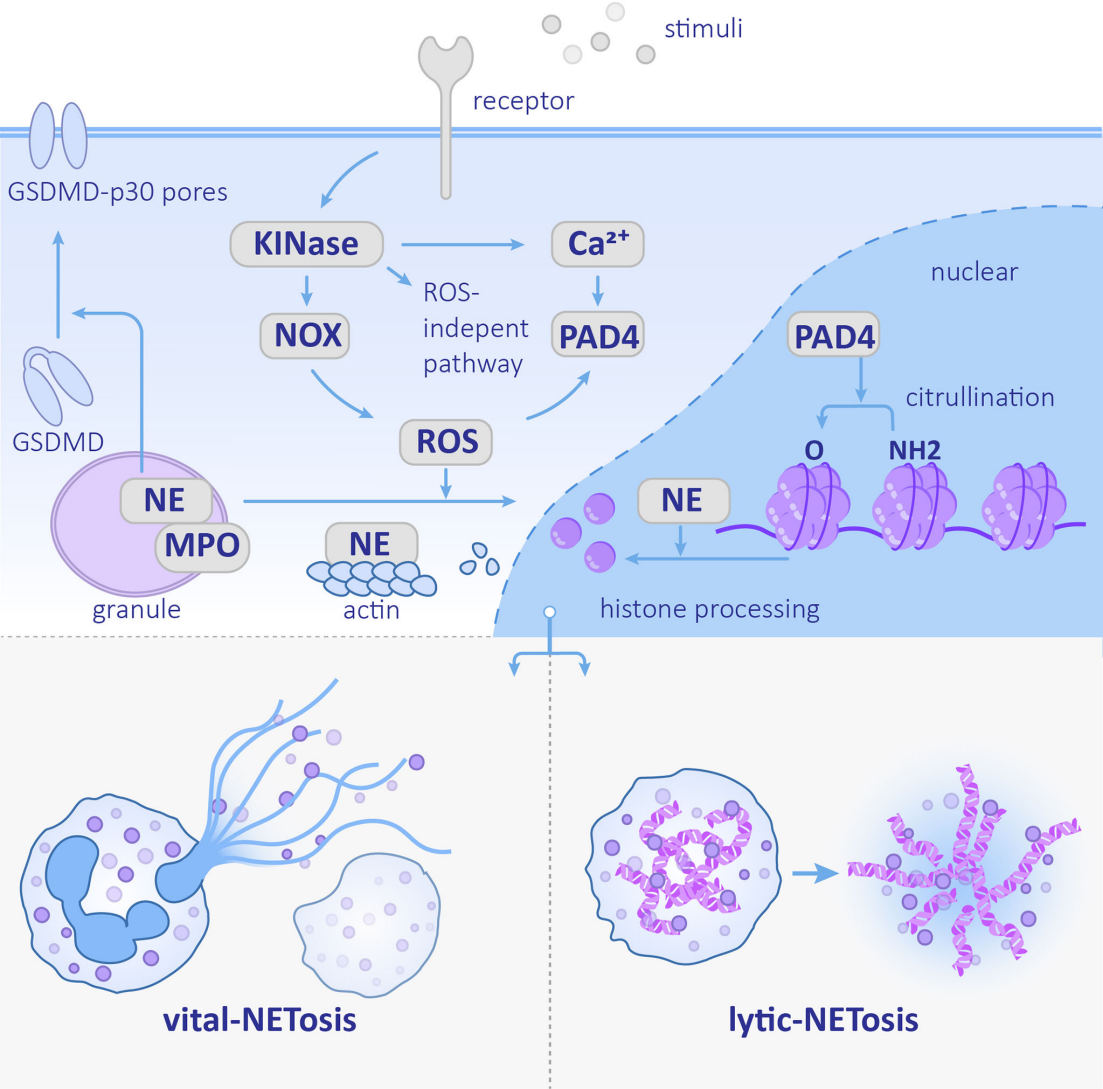
Neutrophil extracellular trap (NET) formation Stimulation recognized by the corresponding receptor activates the downstream kinase and ROS pathway or ROS-independent pathway. NE nuclear transfer is promoted by ROS and helps degrades histones. PAD4 promotes histone citrullination. The NETs formation can be divided into vital-NETosis and lytic-NETosis. NE, neutrophil elastase; PAD4, protein-arginine deiminase type 4; MPO, myeloperoxidase; NOX, NADPH oxidase; ROS, reactive oxygen species.

The process of NET formation is called NETosis. NETosis is a type of programmed cell death. However, it is currently believed that not all NETs formation ends up with cell death (Boeltz et al., 2019). The cell death-involved one is called lytic NETosis, and the other one is termed vital NETosis as shown in **Figure 1** (Papayannopoulos, 2018). Lytic NETosis usually occur within 3 to 8 h of neutrophil activation. This process includes actin remodeling, cell depolarization, nuclear membrane disappearance, chromosome decondensation, and an improvement in cell membrane permeability. However, vital NETosis induced by *Staphylococcus aureus* occurs within a few minutes, which is much more rapid than lytic NETosis. The chromatin is secreted out of the neutrophils and the remaining nuclear-free cells can still perform phagocytosis (Pilsczek et al., 2010). During vital NETosis, mitochondrial DNA (mtDNA), rather than nuclear DNA, is excreted by neutrophils that have been stimulated by granulocyte/macrophage colony-stimulating factor (GM-CSF), toll-like receptor 4 (TLR4) stimulation, or complement factor 5a (C5a) receptor stimulation (Yousefi et al., 2009). Unlike nuclear DNA that physically traps pathogens, mtDNA has additional proinflammatory effects (Lood et al., 2016; De Gaetano et al., 2021).

A variety of stimuli, such as phorbol 12-myristate 13-acetate (PMA), calcium ionophores, pathogenic microorganisms, and immune complexes, can trigger the release of NETs. Stimuli influence both the structure and morphology of NETs. PMA- and *Pseudomonas aeruginosa*-induced NETs are cloud-like structures, while *C. albicans*- and *Staphylococcus aureus*-induced NETs consist of elongated filaments (Sosa-Luis et al., 2021). Besides, the components involved in NETosis vary depending on stimuli (Kenny et al., 2017).

Reactive oxygen species (ROS) are the protagonists of NETosis. The upstream ROSs are dependent on different stimuli-dependent kinases, such as protein kinase C, extracellular-signal-regulated kinase, phosphoinositide 3-kinase, and IL-1 receptor-associated kinase (PKC, ERK, PI3K, and IRAK, respectively) (Hakkim et al., 2011; DeSouza-Vieira et al., 2016; Zawrotniak et al., 2017). The involvement of PKC was primarily discovered in NEToisis induced by PMA, which is a non-physiological stimulus that is used widely in research. PKC is also required for *C. albicans*- and group B *Streptococcus* (GBS)-induced NETosis (Kenny et al., 2017). Besides, syk is also an important kinase, especially in *Candida* species-induced NET formation (Clark et al., 2018). A syk-deficient neutrophil lacks fungicidal activity due to a decline in ROS production and NET formation (Negoro et al., 2020).

The kinases mentioned above phosphorylate the NADPH oxidase (NOX) subunit gp91phox/Nox2 and catalyze ROS production, which can stimulate the downstream MPO–NE pathway. MPO forms a complex with NE that stretches across the azurophilic granule membrane. After MPO is stimulated by ROS and produces oxidants, nuclear translocation of NE is promoted. NE eventually acts on histones to assist chromosome de-condensation (Papayannopoulos et al., 2010). Additionally, NE cleaves Gasdermin D (GSDMD) and forms GSDMD-p30 pores in the plasma membrane (Sollberger et al., 2018).Finally, the nucleus disintegrates, the plasma membrane ruptures, and NETs are released (**Figure 1**).

Unlike NE, other neutrophil serine proteases (NSPs) stored in azurophilic granules, including Cathepsin G (CatG), proteinase 3 (PR3), and NSP4, do not play a role in NET formation. These NSPs exist in NETs in an inactive conformation; therefore, they have no antimicrobial functions (Kasperkiewicz et al., 2020). In addition to NE, the de-condensation of chromosomes also depends on the histone H3 citrullination. This reaction is catalyzed by protein-arginine deiminase type 4 (PAD4), which can be activated by the high concentration of calcium (Li et al., 2010). In addition to chromatin de-condensation, PAD4 is also required for nuclear envelope rupture and extracellular DNA release (Thiam et al., 2020). Whereas PAD4 is not indispensable in every kind of NETosis (Holmes et al., 2019), the relative importance of NE and PAD4 is associated with the stimulus. NE is more crucial in *C. albicans*-induced NETosis, while PAD4 plays a more important role in calcium ionophore- and bacteria-induced NETosis (Li et al., 2010; Lewis et al., 2015) as shown in **Figure 1**.

However, ROS is not always required for NET formation. Chronic granulomatous disease (CGD) is found in neutrophils that are deficient in NADPH oxidase activity are often used as a tool to study the role of ROS in NETosis (Yu et al., 2021). Since some CGD patients have residual NADPH oxidase activity, it is necessary to measure ROS production initially. After comparing the number of NETs formed by healthy neutrophils versus CGD neutrophils, results demonstrated that ROS is necessary for PMA-induced NET formation, while *C. albicans*- and GBS-induced NETosis are only partially required, and no requirement for calcium and potassium ionophores are necessary (Kenny et al., 2017). The molecular mechanisms of ROS-independent NETosis remain unclear. It was recently found that the calcium-induced ROS-independent NETosis is mediated by the SK3 channel (a family member of calcium-activated small conductance potassium channels) and mitochondrial ROS. Hypercitrullination of histone H3 is also involved in this process (Pathakumari et al., 2020).

## NET formation triggered by fungal pathogens and NET fungicidal effect

**Figure 2:**
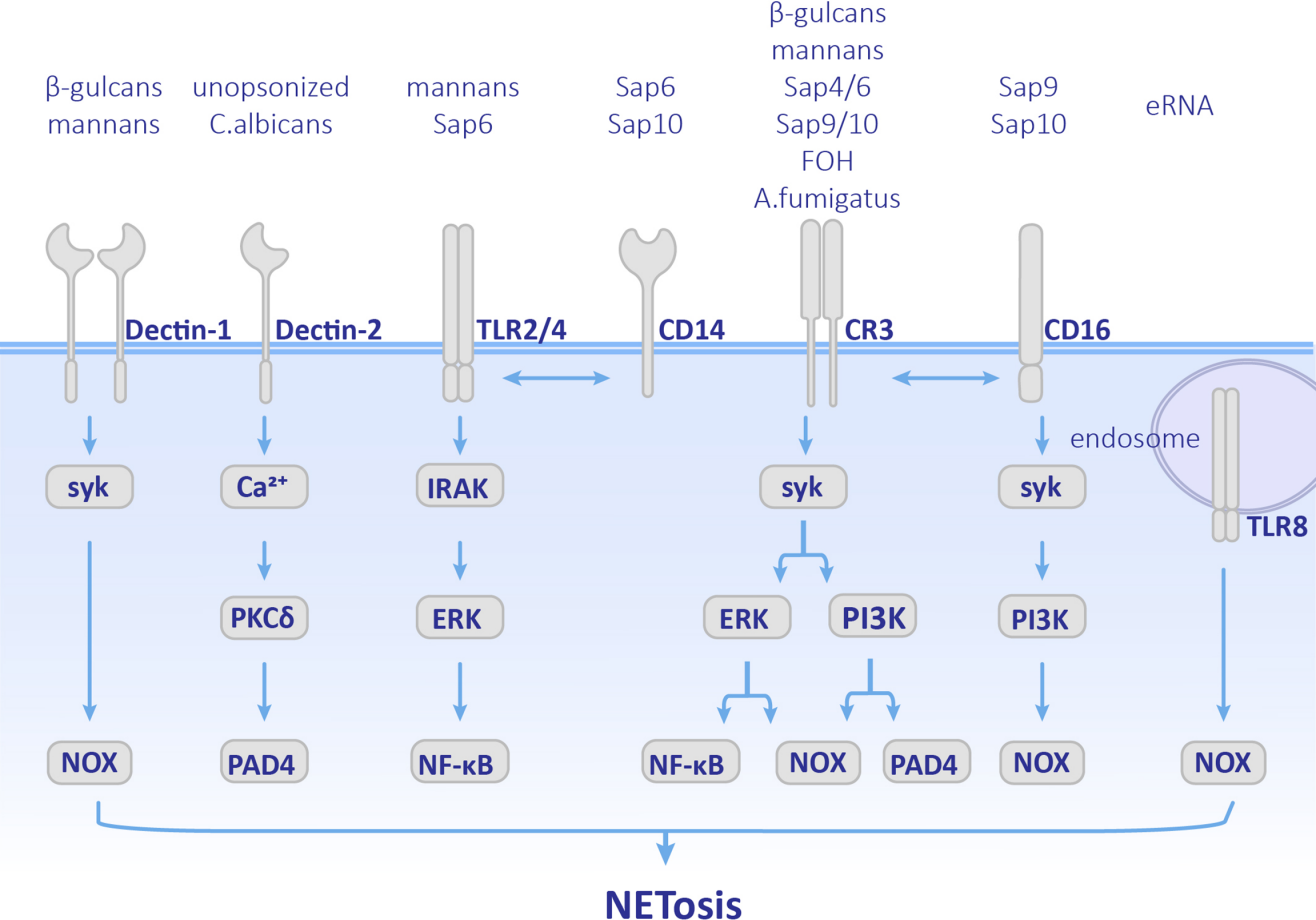
Pathways employed by different fungal components. Cellular components involved in NETosis vary depending on different fungal stimulation. Neutrophils recognize by Dectin-1, Dectin-2, TLRs, CD14, CD16 and CR3 receptors, and activate both ROS pathway and ROS-independent pathway. CR3, complement receptor 3; TLR, Toll-like receptor; MPO, myeloperoxidase; ROS, reactive oxygen species.

NETosis has been discovered in many mycoses. It can be induced by diverse fungal components and fungal secreted proteins. It is generally believed that patterns [pathogen-associated molecular patterns (PAMPs)] on the surface of fungal cell walls, which can be recognized by pattern recognition receptors (PRRs) on neutrophils result in NET formation. The following section summarizes NETosis pathways stimulated by various fungal stimulation (**Figure 2**) and discusses the role of NETs in innate immunity against fungal pathogen.

### Candida albicans

*C. albicans* is a common opportunistic pathogen leading to skin and mucous membranes or systemic infections, especially in immunocompromised patients. *C. albicans* is polymorphic. *C. albicans* conidia normally have a symbiotic relationship with the host. Once they transform into the hypha form, their virulence is enhanced and even results in disseminated infections (Shepherd et al., 1985; Cleary et al., 2016). *Candida* yeast can be phagocytized by neutrophils, while the hyphae are too large to be engulfed. As early as 2006, it was observed that *C. albicans* could induce NET formation (Urban et al., 2006). Later on, NETosis was found in both mice and zebrafish *C. albicans* infection models in which they showed antifungal activity (Ermert et al., 2009; Gratacap et al., 2017). Previous research has mainly focused on opsonized *C. albicans*, which undergoes complement receptor 3 (CR3) (CD11b/CD18)-mediated NET formation *via* the Syk-PKC-ROS pathway (Kenny et al., 2017; Wu et al., 2017). PI3K is also involved in this pathway. Inhibition of PI3K results in a decrease in ROS production and NE nuclear transfer and finally leads to less NETosis (Romao et al., 2015). PAD4-mediated histone H3 citrullination occurs during NET formation, but inhibition of PAD4 does not affect the release of NETs (Guiducci et al., 2018). Therefore, PAD4 is not indispensable in opsonized *C. albicans*-induced NETosis. The role of unopsonized *C. albicans* has also been supplemented recently. Unopsonized *C. albicans* can induce rapid NET release through the ROS-independent Dectin-2-mediated Syk-Ca^2+^–PKCδ–PAD4 pathway (Wu et al., 2019) as shown in **Figure 2**.

Recently, the molecular mechanisms used d by specific components of *C. albicans*, such as cell wall and biofilm components, have been further studied (**Figure 2**). Mannans and β-glucans were found to be cell wall polysaccharides of *C. albicans*, which are crucial PAMPs. Mannans can be recognized by CD14, TLRs, and Dectin-1, and promote the formation of NETs through ROS-independent pathways, while β-glucan is recognized by receptors Dectin-1 and CR3 and induce NETosis (Li et al., 2010). Researchers have also found that when the extracellular matrix component fibronectin (Fn) is present, β-glucans on *C. albicans* hyphae can be recognized by CR3 and induces rapid ROS-independent release of NETs. This process depends on the extracellular signal-related kinase/mitogen-activated kinase (ERK/MAPK) pathway (Byrd et al., 2013) and suggests that extracellular matrix components are also involved in the regulation and induction of NET formation. In addition, recognition between Fn+β-glucan and CR3 has crosstalk with β1 integrins, VLA5 and VLA3 (α5β1 and α3β1, respectively). First CR3 promotes NETosis by activating VLA5, and then CR3 inside-out self-activation happens, leading to both VLA5 inhibition and VLA3 activation after which crosstalk between CR3 and β1 integrins first causes acceleration of NETosis, and second leads to inhibition of NET formation and promotion of the neutrophil accumulation at the location at which NETosis has occurred (Johnson et al., 2017). Unlike mannans and β-glucans, Als3 (a member of the agglutinin-like sequence family) and enolase, which are cell wall surface proteins of *C. albicans* did not stimulate NETosis (Zawrotniak et al., 2017). However, they can bind to proteins in NETs such as MPO, NE, lactotransferrin (LF), and LL-37 (cathelicidin-derived peptide) (Karkowska-Kuleta et al., 2021).

*C. albicans* forms biofilms on the surface of host tissues to protect fungal cells. The phenomenon that microorganisms gathered in biofilms communicate with each other is called quorum sensing (QS). The molecules that play a role in QS are named quorum sensing molecules (QSMs) (Padder et al., 2018). QSMs are composed of farnesol (FOH), farnesolic acid (FA), and tyrosol (TR). Among them, FOH, which is sesquiterpene alcohol released by *C. albicans* outside of the cell, is a chemotactic factor for neutrophil aggregation. Moreover, FOH can trigger NET formation. Neutrophils recognize FOH through CR3 and TLR2 and promote ROS-dependent NETosis (Zawrotniak et al., 2019). Furthermore, the nucleic acid components in *C. albicans* biofilms can also induce NETosis. Extracellular RNA (eRNA) is released into the biofilm by *C. albicans*, which can enhance the biofilm’s resistance to antifungal drugs, and eDNA is recognized by TLR8 and promotes NETosis in ROS-dependent manner (Smolarz et al., 2021).

*C. albicans* modulates NET formation mainly *via* the expression of virulence factors and morphological transformation. Secretory aspartic proteases (Saps) are virulence factors produced by *C. albicans* and have a chemotactic effect on neutrophils (Gabrielli et al., 2016). Different members of Saps induce different pathways and levels of NET formation. Saps4 and 6 can cause the strongest release of NETs, which is induced by both ROS-dependent and -independent pathways after being recognized by the CD11b receptor. Saps9 and 10 induce the formation of NETs through ROS-dependent pathways after being recognized by CD16 and CD18 receptors. Saps6 and 10 also stimulate CD14, which is the co-receptor of TLR4 (Gabrielli et al., 2016) as shown in **Figure 2**. Phospholipase is another virulence factor of *C. albicans*. Researchers have found strains of *Candida* spp. that produced phospholipases could induce NETosis, while *C. glabrata* did not (Campos-Garcia et al., 2019). Since this research did not control other variables between *Candida* spp., the role of phospholipases in NETosis needs further investigation.

The morphological transformation of *C. albicans* has a great influence on NET formation. Hyphae- and yeast-induced NETosis differ in amount, kinetics, and mechanisms (Kenno et al., 2016). For instance, autophagy is involved in different periods of hyphae- and yeast-induced NETosis. It is currently believed that the formation of NET is related to autophagy-mediated DNA unfolding and excretion (Remijsen et al., 2011). In both forms of *C. albicans*, autophagy plays a role in the rapid induction of NETs, while the yeast form uses both autophagy and ROS pathways. When exposed to neutrophils for 4 h, only the hyphae form can induce NETosis through autophagy and the ROS pathway (Kenno et al., 2016). However, whether rapid NET formation caused by *C. albicans* as mentioned above is of the same type as the rapid vital-NETosis induced by *Staphylococcus aureus* needs further study (Pilsczek et al., 2010; Byrd et al., 2013; Kenno et al., 2016). When compared with hyphae, *C. albicans* conidia has more difficulty inducing the release of NETs. This size-dependent NET formation is regulated by Dectin-1, which acts as a phagocytic receptor. Activation of Dectin-1 abrogates NE nuclear transmission and sequesters NE in phagosomes leading to phagocytosis rather than NETosis (Branzk et al., 2014). Therefore, Dectin-1 induces contradictory effects in NET formation. In addition, differences in neutrophil responses induced by different clinical isolates of *C. albicans* have been found. The most commonly used strain in research is SC5314, and it differs from other strains in terms of the genome, toxicity, resistance to immune cell killing (Hirakawa et al., 2015), and interactions with neutrophils, which includes the induction of neutrophilic ROS production, NET release, phagocytosis, and tumor necrosis factor (TNFα) production (Shankar et al., 2020). Compared with 3683 strains, SC5314 induces more NETs, a finding that can be explained by higher expression of Rac2 (Zhang et al., 2017), which is the member of the Rho family GTPases that participate in the formation of NETs (Lim et al., 2011). Moreover, compared with pseudohyphal isolates P78042 and P57072 and yeast isolates P94015, SC5314 can prompt neutrophils to release the largest amount of NETs, which is directly proportional to the degree of hyphae formation (Shankar et al., 2020).

Both *C. albicans* conidia and hyphae can be captured or entwined by chromatins in by NETs and then killed by the granular proteins (Urban et al., 2006; Karkowska-Kuleta et al., 2021), among which calprotectin is essential for the elimination of *C. albicans* (Urban et al., 2009). Of note, MPO is also a key antifungal factor in NETs. Patients with severe congenital neutropenia (SCN) have mutations in the gene for jagged 1 protein, (JAGN1). When JAGN1 expression-inhibited neutrophils are co-cultured with *C. albicans*, the production of MPO in NETs was found to be reduced, and the antifungal activity declined (Khandagale et al., 2018). Neutrophils from MPO-deficient patients are weak in NET-dependent *C. albicans* growth inhibition (Metzler et al., 2011). Furthermore, attention should be paid to the finding that NETs can also influence fungal cell wall epitope changes. NETs function in unmasking β-glucan and enhancing Dectin-1 recognition in a ROS-dependent manner, which leads to an elevation in the interleukin 6 (IL-6) response and promotion of fungal containment. However, proteases, such as NE, cathepsin G, and proteinase 3, do not have a role in triggering cell wall remodeling (Hopke et al., 2016). Further investigation is required to elucidate the exact NET component that mediates this process.

Another intriguing study points out a negative aspect of NETs, which is the destruction of human tissue associated with interacting with *C. albicans*. Protein components of NETs, such as NE, MPO, lactotransferrin, and LL-37 (cathelicidin-derived peptide), binding with *C. albicans* surface proteins results in surrounding tissue damage and acceleration of fungal invasion (Karkowska-Kuleta et al., 2021). Moreover, NET-dependent tissue damage and fungal invasion can be promoted by Saps, which is secreted by *C. albicans*. Saps play a role in the cleavage of α1-proteinase inhibitor (A1PI), which is a serine protease inhibitor that can inhibit NE and lead to restoration of NE, which is a crucial part of NET-dependent tissue damage (Gogol et al., 2016).

### Aspergillus fumigatus

*Aspergillus fumigatus* is an important saprotrophic fungus with a strong capacity to resist human immune system attacks. The lung is the most common site of infection and leads to pulmonary aspergillosis (Latgé and Chamilos, 2019). Both conidia and hyphae trigger the formation of NETs in human neutrophils (McCormick et al., 2010). Morphotypes and strains of *A. fumigatus* have a decisive impact on the formation of NETs. Swollen and resting conidia, especially, result in less NET production. When compared with the ATCC46645 wild-type strain, DAL wild-type strain showed weaker NET inducibility (Bruns et al., 2010). Additionally, NETs were observed as the first immigrating neutrophils 3–4 h after infected with *A. fumigates* (Clark et al., 2016).

ROS pathway is involved in *A. fumigatus*-induced NETosis. This process is mediated by CR3, among which the I domain of CD11b has a recognition function, and activated downstream Syk-PI3K pathway. PDA4 is also involved, but PAD4 does not affect the fungicidal capability of the NETs (DeSouza-Vieira et al., 2016). Addition of the PDA4 inhibitors, Cl-amidine and GSK484, did not lead to cessation of *A. fumigatus*-induced NETosis (Silva et al., 2020). Moreover, the ROS-independent pathway is also used by *A. fumigatus*. This finding is supported by studies using neutrophils from CGD patients (Gazendam et al., 2016). Nevertheless, the pathway that promotes ROS-independent NETosis is still unknown.

The effect of NETs on *A. fumigatus* is controversial. On the one hand, some believe that NETs are capable of inhibiting the germination of *A. fumigatus* conidia and killing the hyphae (Bruns et al., 2010; Gazendam et al., 2016). The fungicidal function is related to the NETs granules components. Calprotectin in NETs can capture the extracellular zinc of *A. fumigatus*, thereby inhibiting the germination of *A. fumigatus* conidia (Gazendam et al., 2016). This inhibitory effect can be reduced by the addition of Ze^2+^ (McCormick et al., 2010). The anti-hyphal activity of calprotectin can be restored by injecting recombinant calprotectin into calprotectin-deficient mice (Clark et al., 2016). Moreover, it was found that pentraxin 3 (PTX3), a secreted pattern recognition molecule with a known nonredundant role in resistance to *A. fumigates*, is localized in neutrophil granules and released into the NETs. NET component proteins, such as MPO and histone are PTX3 ligands, which can be enriched by PTX3 around PTX3-captured pathogens and contribute to host defense (Garlanda et al., 2002; Jaillon et al., 2007; Daigo and Hamakubo, 2012; Daigo et al., 2016). On the other hand, other researchers hold opposite opinions. The addition of DNAse during the formation of NETs does not abrogate the antifungal capability of neutrophils, which suggests that inhibition of germination is mainly due to phagocytosis (McCormick et al., 2010; Gazendam et al., 2016). Therefore, the above results indicate that NETs can control *A. fumigatus* infection to a certain extent even though they are not adequate enough to eradicate *A. fumigatus.* Besides, another study shows that NETs in invasive pulmonary aspergillosis reduce fungal clearance and increase tissue damage (Alflen et al., 2020). However, this study used PAD4-deficient mice as the experimental group and citrullinated H3 as an indicator of NETs formation. This method contradicts the view that PAD4 is not necessary for *A. fumigatus* to induce NET release (Silva et al., 2020); thus, further research is still needed.

### Other fungi

*Histoplasma capsulatum* can also induce NET formation. This fungus activates Scr and Syk through a ROS-dependent pathway mediated by CD18 instead of Dectin-1, and eventually, NETosis occurs. Similarly, PAD4 participates in this process, but it is dispensable (Thompson-Souza et al., 2020).

While *Paracoccidioides brasiliensis* is mainly recognized by Dectin-1 receptors, the network structure of the NETs can inhibit the spread of *P. brasiliensis* (Bachiega et al., 2016). Two strains of *P. brasiliensis*, Pb18 (a virulent strain) and Pb265 (a less virulent strain) can cause the release of NETs *in vitro*, and the presence of NETs has been found in skin lesions (Della Coletta et al., 2015). NETs also have a killing effect on *P. brasiliensis* because the antifungal activity of neutrophils after DNAse treatment decreases (Bachiega et al., 2016). This finding contradicts the results of a previous study in which neutrophils were co-cultured with *P. brasiliensis* treated with DNAse. No decrease in the number of colony forming units (CFUs) was observed. Therefore, it is believed that NETs are ineffective against *P. brasiliensis* (Mejía et al., 2015). This contradiction may be due to differences in the research methods used to investigate neutrophil antifungal activity. The former study used granulocyte macrophage colony-stimulating factor (GM-CSF), interferon gamma (IFNγ), and TNFα to stimulate neutrophils to simulate the situation of neutrophil activation *in vivo*, while the latter did not.

*Phialophora verrucosa* is the pathogen of chromoblastomycosis. Both opsonized and unopsonized conidia of *P. verrucosa* can induce NET production, while the opsonized one was found to induce more. This process is dose-dependent. The higher the proportions of conidia, the more NETs are induced. NETs can capture and kill both hyphae and conidia of *P. verrucosa* (Liu et al., 2021).

*Scedosporium apiospermum* is an opportunistic pathogen, and the most common site of infection is the lung. NETs have also been found in the lungs of mice infected with *S. apiospermum*. NETs can exert antifungal activity in the early stages of *S. apiospermum* lung infection (Luna-Rodríguez et al., 2020).

## Fungal escape from NETs

**Figure 3:**
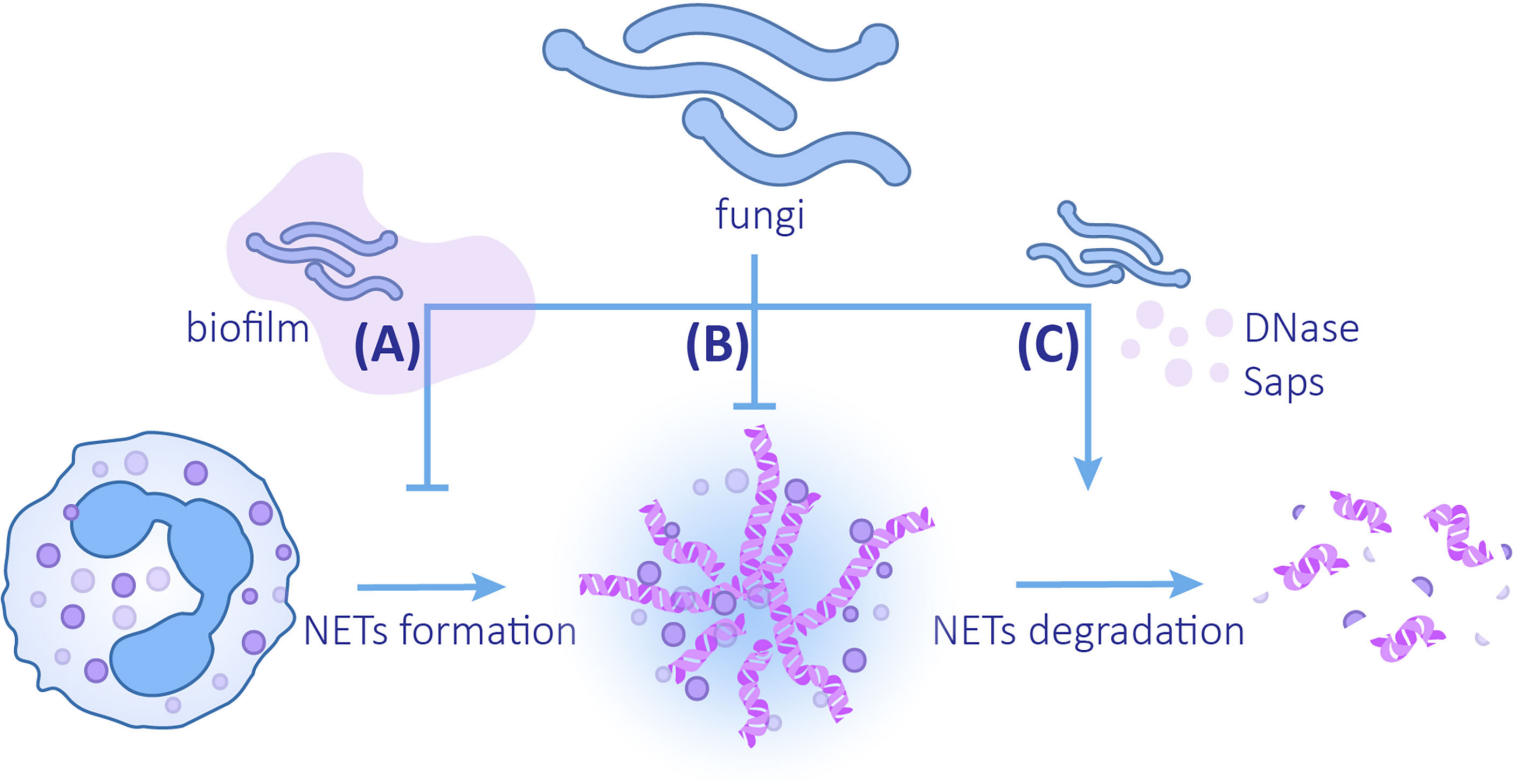
The mechanisms of fungi evading NETs **(A)** Fungi can reduce the release of NETs by forming biofilms. **(B)** Changes in fungal cell wall compositions and biofilm formation can enhance the ability to resist NETs. **(C)** Fungi can promote the degradation of NETs by secreting DNAse and Saps.

Three mechanisms for microorganisms to evade the killing of NETs are known: (1) inhibition of the formation of NETs, (2) formation of capsules and biofilms to enhance resistance to NETs or avoid interaction with NETs, and (3) secretion of proteins to degrade NETs (Papayannopoulos, 2018; Ríos-López et al., 2021). Fungal evasion of NETs also follows the above strategies (**Figure 3**).

### Fungi cause a reduction in NET formation and improve the capability to resist NETs

Biofilms provide a protective extracellular matrix for *C. albicans*, which can not only resist the killing effect of NETs but also reduce the formation of NETs. When compared with planktonic *C. albicans*, ROS production and release of NETs induced by *C. albicans* biofilm is reduced (Kernien et al., 2020). This inhibition depends on the intact biofilm coming into contact with the NETs. The destroyed biofilm or the supernatant of biofilm does not inhibit NETs formation, which implies that soluble substances in biofilm do not exert inhibitory function (Johnson et al., 2016). Even though different clinical isolates produce biofilms varying in thickness and structure, they all attenuate the formation of NETs. Most isolates lead to a decrease in ROS generation. In contrast, 98–210 strains elicit production of ROS despite the inhibitory effects of ROS on NETosis (Kernien et al., 2017). Analogously, the biofilm of *C. glabrata* also has a defensive effect on NETs. Compared with planktonic *C. glabrata*, the *C. glabrata* biofilm induces a decrease in neutrophil ROS production and NET formation (Johnson et al., 2017).

In addition to biofilms, fungal cell wall components are the earliest elements that come into contact with neutrophils. Hydrophobin RodA was the first discovered cell wall component that inhibits *A. fumigatus*-induced NETosis. This protein binds to the cell wall polysaccharide by glycosylphosphatidylinositol (GPI) anchor proteins and forms a coating on the surface of conidia. In this case, PAMPs, such as mannan and β-glucan, are covered and hard to recognize (Carrion et al., 2013). RodA is expressed on conidia rather than hyphae, which explains why conidia trigger less NETosis; therefore swollen or resting conidia with *rodA* mutation causes a drastic increase in the release of NETs in contrast to the wild type (Bruns et al., 2010). The cell wall component, galactosaminogalactan (GAG), also plays a role in resisting NETosis. GAG is an extracellular polysaccharide that contains galactose and n-acetylgalactosamine (GalNAc) (Fontaine et al., 2011). Studies have found that when compared with the more virulent *A. fumigatus*, *A. nidulans*’ GAG contains less GalNAc and has an incomplete biofilm. When UDP-glucose 4-epimerases uge3 or ugeB is overexpressed leading to an increase in the GalNAc content in GAG, biofilm formation of *A. nidulans* is promoted, and the capability to defend against NETs is improved (Lee et al., 2015). This process may be due to the deacetylation of GalNAc, which leads to a positively charged GAG, thereby inhibiting the positively charged antimicrobial peptides and histones in NETs from coming into contact with fungi (Lee et al., 2016).

The capsule is a common factor protecting bacteria against NETs (Wartha et al., 2007), which is rare in fungi. Nevertheless, *Cryptococcus neoformans* is a distinctive fungal pathogen with a coating (the main virulence factor) over its polysaccharide capsule. The predominant component of the polysaccharide capsule is glucuronoxylomannan (GXM), while glucuronoxylomannogalactan (GXMGal) occupies only a small portion of the capsule (Heiss et al., 2009; Fonseca et al., 2019). It has been found that only acapsular strains, CAP67 and GXMGal, can trigger NETosis *via* an ROS-dependent pathway, while GXM cannot, a finding that implies that GXM is important for defense against NETs (Rocha et al., 2015).

Another fungus of the genus Candida, *C. auris*, is an emerging pathogen that threatens global public health (Lone and Ahmad, 2019). Compared with *C. albicans*, *C. auris* is more resistant to neutrophils. No NETs and less ROS production were found in the human neutrophils co-cultured with *C. auris* or in a zebrafish model of invasive candidiasis. Citrullination of histones was not observed either (Johnson et al., 2018). However, *C. auris* cannot inhibit ROS production or PMA-induced NET formation, so *C. auris* does not directly inhibit the ROS pathway to reduce NET production (Johnson et al., 2018).

The oxygen content in the environment also affects the resistance of fungi to NETs. A hypoxic environment can occur at the infection site in which the phagocytosis of neutrophils, the capability of producing ROS, and the capability of releasing NETs are reduced. Hypoxia improves the escape capability of *C. albicans*. This finding may be due to hypoxia that causes *C. albicans* cell wall masking, which prevents β-glucan from being recognized by neutrophil receptors (Lopes et al., 2018).

### Fungi degrade NETs

Fungi can accelerate the degradation of NETs by producing enzymes. It was revealed that DNase assists microbes in evading NETs by directly degrading the DNA components (Buchanan et al., 2006). An extracellular nuclease secreted by *C. albicans* and *C. glabrata*, 3’-nucleotidase/nuclease (3’NT/NU), has a degrading effect on NETs. Adding the 3’NT/NU inhibitor, ammonium tetrathiomolybdate (TTM), to the neutrophils co-cultured with both *C. albicans* and *C. glabrata* can lead to a reduction in the destruction and degradation of NETs (Afonso et al., 2021). DNase secretion is observed in both *C. albicans* SC5314 and 3683 strains; however, DNase production of strain SC5314 is greater (Zhang et al., 2017). Of note, it has been suggested that virulent strain *P. brasiliensis* Pb18 may use the same method to degrade NETs. NETs induced by the two strains of *P. brasiliensis*, Pb18 (a virulent strain) and Pb265 (a less virulent strain), have morphological differences. The NETs induced by Pb265 are denser, while the NETs induced by Pb18 are looser and larger in area, which is similar to the degraded form (Della Coletta et al., 2015). Subsequent studies confirmed this view by using DNase TEST Agar. When compared with Pb265, virulent strain Pb18 co-cultured with neutrophils has a higher expression of PADG_08528, which is a hypothetical protein in the fungus genome (Desjardins et al., 2011), suggesting that this gene may be related to the production of DNAse-like protein (Zonta et al., 2020). In addition to degrading chromatins, degrading NETs proteins by Saps secreted by C.albicans is another available method (Rapala-Kozik et al., 2015; Gogol et al., 2016; Karkowska-Kuleta et al., 2021). LL-37 and histones are most sensitive components to Saps, especially Saps3 and Saps9, which are most effective in NETs proteins degradation and weakening NETs (Rapala-Kozik et al., 2015; Karkowska-Kuleta et al., 2021).

## NETosis provides new ideas for clinical antifungal strategies

Unraveling the mechanisms of the fungi-NETs interactions will help provide new ideas for treating fungal infections. For example, restoration of NADPH oxidase activity in GCD patients with refractory *A. nidulans* lung infection using gene therapy shows a remarkable curative power. After gene therapy, NET formation can be observed in GCD neutrophils, which leads to inhibition of *A. nidulans* growth (Bianchi et al., 2009). This antifungal activity of NETs is calprotectin-dependent (Bianchi et al., 2011). This discovery highlights the consequence of metal chelation in defending against fungal infections. Besides, treating *C. albicans* biofilms with sub-inhibitory concentrations of echinocandin can increase the release of NETs and weaken the protective effects of biofilms. Echinocandin’s effects may be due to the promotion of *C. albicans* cell wall remodeling in which β-glucan on the cell wall is exposed, thereby stimulating the NET release (Hoyer et al., 2018). The latest study found that extracelluar traps might be trained as a memory response termed “trained immunity” (Gao et al., 2022), which refers to the phenomenon that certain pathogens promote stronger responses of innate immunity against reinfection (Netea et al., 2016). The formation and killing capacity of extracellular traps can be heightened by *C. albicans* (Gao et al., 2022). It implicates that pre-treatment of *C. albicans* may benefits infectious diseases and targeting extracellular traps memory responses is a prospective antifungal strategy. However, excessive NET formation induced by trained immunity results in becteriemia and endoxemia (Vitkov et al., 2021). Therefore, modulating the memory response of NETs to treat fungal infections is still challenging. Many pathways for fungi to promote the release of NETs or to evade the killing of NETs exist, all of which provide rich targets for antifungal therapy and may help solve the problem of antifungal drug resistance.

Furthermore, NET formation may be a prospective indicator for prognosis and diagnosis. In *C. albicans* keratitis, the larger the amounts of NETs found in corneal scraps is, the better the treatment effect is. It is a pity that there no prominent difference between NETs from bacterial keratitis and fungal keratitis can be detected; therefore, the diagnostic function of NETs still requires more studies (Jin et al., 2016). To push the prognostic and diagnostic function of NETs forward, it is also important to optimize detection and quantification methods. Computational methods, such as support vector machines and convolutional neural networks, help assess NETs detected by flow cytometry and confocal microscopy (Ginley et al., 2017; Zharkova et al., 2019).

In addition, fungi-induced NETosis may offer medication guidance in clinical practice. People who use biologics, chemotherapeutics, and glucocorticoids are more susceptible to fungal infections (Chen et al., 2017; Fan et al., 2020), owing to immune system suppression. The latest research found that this suppression is related to the inhibition of NETs. In *C. albicans* keratitis, glucocorticoids not only lead to an increase in the virulence of the fungus but also promote disease progression by limiting NET formation (Fan et al., 2020). Notably, not all immunodeficiency populations’ vulnerability to fungus is caused by low NETs. Emerging *Candidas*-induced neonatal sepsis may be associated with deficiencies in neutrophil phagocytosis and respiratory burst (Melvan et al., 2010) rather than NETosis because neonatal neutrophils show active CR3-mediated rapid NETosis after encountering *C. albicans* (Byrd et al., 2016). Interestingly, some antifungal drugs also have inhibitory effects on NETs. Traditional amphotericin B, liposomal amphotericin B, and voriconazole can inhibit the release of NETs by neutrophils co-cultured with inactivated *A. fumigatus* hyphae (Decker et al., 2018). The influence of this phenomenon on the effects of antifungal drugs remains to be studied. This finding shows the potential to be served as a guide for clinical antifungal usage.

Since overwhelming NET formation may result in tissue damage, especially in invasive pulmonary aspergillosis (Alflen et al., 2020), it is important to maintain the balance and determine the mechanisms used by NETs in causing collateral damage. Components of NETs, such as histones and MPO, produce direct cytotoxic effects on epithelial and endothelial cells (Saffarzadeh et al., 2012). Besides, proteins in NETs such as MPO, NE, lactotransferrin (LF), and LL-37 can directly bind to proteins on *C. albicans* cell wall like Als3, enolase and Gmp1. *C. albicans* hyphae covered by these proteins have stronger destructive power to epithelial cells and promote fungal invasion (Karkowska-Kuleta et al., 2021). Recent studies have found that NETs can promote macrophage polarization and pyroptosis, which induces inflammation following infections (Chen et al., 2018; Song et al., 2019). In turn, different polarized macrophages are responsible for modulating NET degradation (Haider et al., 2020). Therefore, several available agents, such as DNase1 and histone and MPO inhibitors can be applied to prevent NETs-associated damage (Block and Zarbock, 2021).

## Perspectives and conclusion

Releasing extracellular traps is a common phenomenon among mononuclear cells and granulocytes (Daniel et al., 2019; Conceição-Silva et al., 2021). Monocytes extracellular traps and macrophage extracellular traps (MoETs and METs, respectively) are observed when cocultured with *C. albicans* and also have fungicidal effects (Liu et al., 2014; Halder et al., 2016; Loureiro et al., 2019a; Loureiro et al., 2019b). Eosinophils extracellular traps (EETs) are found in the bronchial secreta of allergic bronchopulmonary aspergillosis (ABPA) patients and lead to an increase in the viscosity of eosinophilic mucus leading to mucus plugging in ABPA and damage to nearby epithelia (Omokawa et al., 2018; Ueki et al., 2018; Barroso et al., 2021). As for mast cells, *C. albicans* can induce mast cells extracellular traps (MSETs), which can only capture fungi cells rather than killing them (Lopes et al., 2015).

NETosis is one form of programmed cell death, and together with apoptosis, necroptosis, and pyroptosis acts as a defense against microorganisms (Jorgensen et al., 2017). Different cell death pathways have mutual effects and form a complicated network (Rosazza et al., 2021). GSDMD used to be considered the central factor of pyroptosis, while it has been found that GSDMD takes part in NETosis. Different proteases, such as NE and caspase-1, are involved in cleavage of GSDMD (Sollberger et al., 2018; Chen et al., 2018). Autophagy assists in chromosome decondensation of NETosis and is required in *C. albican* induced-NETosis (Remijsen et al., 2011; Kenno et al., 2016). The role of cell death in fungal infection and their cross-talk remain to be studied.

Taken together, the mutual effects between fungi and NETs play a vital role in fungal pathogenesis (**Table 1**). Fungi can trigger release of NETs by neutrophils, which shows different killing effects facing different types of fungi. At the same time, fungi can in turn avoid the fungicidal activity of NETs using multiple avoidance mechanisms. The ubiquity of extracellular reticular structure among innate immune cells exhibits a new extracellular mechanism for resisting microbes. This fierce fight between microbes and the immune system shows tremendous opportunities for development of therapeutic agents to treat fungal infections.

**Table 1:** Summary of different fungi interactions with neutrophil extracellular traps (NETs).

Fungal species	Receptors	Pathway	Susceptibility to NETs	Escape mechanism	Refs
***Candida albican***	CR3, Dectin-1, Dectin-2, TLRs, CD14, CD16	mixed	++	Biofilm, DNase, Saps	(Zawrotniak et al., 2019; Urban et al., 2006; Urban et al., 2009; Metzler et al., 2011; Byrd et al., 2013; Romao et al., 2015; Gabrielli et al., 2016; Kenno et al., 2016; Gogol et al., 2016; Johnson et al., 2016; Kenny et al., 2017; Zawrotniak et al., 2017; Wu et al., 2017; Johnson et al., 2017; Guiducci et al., 2018; Padder et al., 2018; Khandagale et al., 2018; Hoyer et al., 2018; Wu et al., 2019; Campos-Garcia et al., 2019; Negoro et al., 2020; Kasperkiewicz et al., 2020; Thiam et al., 2020; Shankar et al., 2020; Kernien et al., 2020; Smolarz et al., 2021; Afonso et al., 2021; Sprenkeler et al., 2022)
***Candida auris***	No NETs were induced		+	ND	(Johnson et al., 2018)
***Candida glabrata***	ND	mixed	+	Biofilm, DNase	(Johnson et al., 2017)
***Aspergillus fumigatus***	CR3	mixed	+	Modulate cell wall components	(Bianchi et al., 2009; McCormick et al., 2010; Bruns et al., 2010; Lee et al., 2015; Clark et al., 2016; Gazendam et al., 2016)
***Aspergillus nidulans***	ND	ND	++	Modulate cell wall components	(Lee et al., 2015)
***Histoplasma capsulatum***	CR3	ROS dependent	++	ND	(Thompson-Souza et al., 2020)
***Paracoccidioides brasiliensi***	Dectin-1	ND	controversial	DNAase	(Della Coletta et al., 2015; Bachiega et al., 2016)
***Phialophora verrucosa***	ND	ND	++	ND	(Liu et al., 2021)
***Scedosporium apiospermum***	ND	ND	+ (erly stage)	ND	(Luna-Rodríguez et al., 2020)
***Capsular neoformans***	ND	ROS dependent	++	Polysaccharide capsule	(Rocha et al., 2015)

CR3, complement receptor 3; TLRs, Toll-like receptors; ND, not determined; ROS, reactive oxygen species.

## Author contributions

CL designed and wrote the manuscript, and finished the illustrations. NL and ML contributed to the specific sub-titles and modification work. All authors contributed to the article and approved the submitted version.

## Funding

This work was supported by the CAMS Innovation Fund for Medical Science (2017-I2M-1-017), the National Natural Science Foundation of China (81773338), and the Nanjing Incubation Program for National Clinical Research Center [2019060001].

## Conflict of interest

The authors declare that the research was conducted in the absence of any commercial or financial relationships that could be construed as a potential conflict of interest.

## Publisher’s note

All claims expressed in this article are solely those of the authors and do not necessarily represent those of their affiliated organizations, or those of the publisher, the editors and the reviewers. Any product that may be evaluated in this article, or claim that may be made by its manufacturer, is not guaranteed or endorsed by the publisher.

## Abbreviations

NETs, neutrophil extracellular traps; MPO, myeloperoxidase; NE, neutrophil elastase; PMA, phorbol 12-myristate 13-acetate; NOX, NADPH oxidase; PKC, protein kinase C; ROS, reactive oxygen species; CR3, complement receptor 3; PAD4, protein-arginine deiminase type 4; CGD, chronic granulomatous disease; PAMPs, pathogen-associated molecular patterns; PRRs, pattern recognition receptors; CR3, complement receptor 3; Fn, fibronectin; FOH, farnesol; eDNA, extracellular DNA; Saps, secretory aspartic proteases; GAG, galactosaminogalactan; GalNAc, galactose and n-acetylgalactosamine; GXM, glucuronoxylomannan; GXMGal, glucuronoxylomannogalactan; 3’NT/NU, 3’-nucleotidase/nuclease.
